# Risk score for first-screening of prevalent undiagnosed chronic kidney disease in Peru: the CRONICAS-CKD risk score

**DOI:** 10.1186/s12882-017-0758-4

**Published:** 2017-11-29

**Authors:** Rodrigo M. Carrillo-Larco, J. Jaime Miranda, Robert H. Gilman, Josefina Medina-Lezama, Julio A. Chirinos-Pacheco, Paola V. Muñoz-Retamozo, Liam Smeeth, William Checkley, Antonio Bernabe-Ortiz, CRONICAS Cohort Study Group, Antonio Bernabé-Ortiz, Juan P. Casas, George Davey Smith, Shah Ebrahim, Héctor H. García, Robert H. Gilman, Luis Huicho, Germán Málaga, J. Jaime Miranda, Víctor M. Montori, Liam Smeeth, William Checkley, Gregory B. Diette, Robert H. Gilman, Luis Huicho, Fabiola León-Velarde, María Rivera, Robert A. Wise, William Checkley, Héctor H. García, Robert H. Gilman, J. Jaime Miranda, Katherine Sacksteder

**Affiliations:** 1 CRONICAS Center of Excellence in Chronic Diseases, Universidad Peruana Cayetano Heredia, Av. Armendariz 497, Miraflores, 18 Lima, Peru; 2 Department of Medicine, School of Medicine, Universidad Peruana Cayetano Heredia, Lima, Peru; 3 Department of International Health, Bloomberg School of Public Health, Johns Hopkins University, Baltimore, USA; 4 Área de Investigación y Desarrollo, Asociación Benéfica PRISMA, Lima, Peru; 5 Universidad Católica de Santa María, Arequipa, Peru; 6 Faculty of Epidemiology and Population Health, London School of Hygiene and Tropical Medicine, London, UK; 7 Division of Pulmonary and Critical Care, School of Medicine, Johns Hopkins University, Baltimore, USA

**Keywords:** Risk assessment, Kidney, Chronic kidney disease, Latin America

## Abstract

**Background:**

Chronic Kidney Disease (CKD) represents a great burden for the patient and the health system, particularly if diagnosed at late stages. Consequently, tools to identify patients at high risk of having CKD are needed, particularly in limited-resources settings where laboratory facilities are scarce. This study aimed to develop a risk score for prevalent undiagnosed CKD using data from four settings in Peru: a complete risk score including all associated risk factors and another excluding laboratory-based variables.

**Methods:**

Cross-sectional study. We used two population-based studies: one for developing and internal validation (CRONICAS), and another (PREVENCION) for external validation. Risk factors included clinical- and laboratory-based variables, among others: sex, age, hypertension and obesity; and lipid profile, anemia and glucose metabolism. The outcome was undiagnosed CKD: eGFR < 60 ml/min/1.73m^2^. We tested the performance of the risk scores using the area under the receiver operating characteristic (ROC) curve, sensitivity, specificity, positive/negative predictive values and positive/negative likelihood ratios.

**Results:**

Participants in both studies averaged 57.7 years old, and over 50% were females. Age, hypertension and anemia were strongly associated with undiagnosed CKD. In the external validation, at a cut-off point of 2, the complete and laboratory-free risk scores performed similarly well with a ROC area of 76.2% and 76.0%, respectively (*P* = 0.784). The best assessment parameter of these risk scores was their negative predictive value: 99.1% and 99.0% for the complete and laboratory-free, respectively.

**Conclusions:**

The developed risk scores showed a moderate performance as a screening test. People with a score of ≥ 2 points should undergo further testing to rule out CKD. Using the laboratory-free risk score is a practical approach in developing countries where laboratories are not readily available and undiagnosed CKD has significant morbidity and mortality.

**Electronic supplementary material:**

The online version of this article (10.1186/s12882-017-0758-4) contains supplementary material, which is available to authorized users.

## Background

Chronic Kidney Disease (CKD) is becoming a health threat globally. CKD ranks among the top 20 causes of years of life lost, and in some countries from the Latin America (LA) and the Caribbean region, it even ranks in the top 10 [1, 2]. Despite these trends, evidence about CKD in low- and middle-income countries (LMICs) is scarce, making it more difficult to assess modifiable risk factors or to identify potential venues for prevention strategies. Recently, a population-based study in two Peruvian cities reported a CKD prevalence of 16.8% [3]. Because of the significant morbidity and mortality of CKD, including cardiovascular disease and dialysis-dependent kidney failure, early detection is especially critical in this context. Thus, given the growing prevalence of CKD, there is a need to identify high risk subjects to prevent a greater burden in terms of morbidity and mortality.

Resources are scarce in LMICs like Peru and other countries in LA challenging the assessment of large populations for CKD. A more practical mean of identifying risk for CKD among individuals is risk scores, because they take together a set of variables and estimate how likely it is for a subject to have a given condition. Although many CKD risk scores have been summarized in a recent review [4], the available evidence suggest some research gaps, including, for example, that none of these risk scores have been developed in populations from LA. Etiologies for CKD are different in LMICs relative to high-income countries. This could explain why people from LA have different CKD prevalence rates when compared to Mexican-Americans, whites and blacks living in the USA [5]. Therefore, risk scores for these populations may not be accurately applied in subjects with Hispanic/Latin background. Many of the risk scores included laboratory-based variables. This is challenging in LMICs where scarce resources also affect the availability of laboratory infrastructure, which are almost unavailable at the primary care level or in rural areas. Some environmental features had not been included, such as geographic location. Living in high altitude has an impact on health because hypoxemia challenges physiological systems, including the kidney [6, 7]. Because globally there are millions of people living over 2000 m (6561 ft) above the sea level [8], results from high-altitude sites could be informative and useful for these populations.

Consequently, we aimed to develop a pragmatic risk score for prevalent undiagnosed CKD, and to assess its performance, in terms of sensitivity, sensibility, positive and negative predictive values, as well as positive and negative likelihood ratio, with and without laboratory-based variables. We used data of two population-based studies conducted in four settings in Peru including subjects living at 3825 m (12,549 ft) above the sea level.

## Methods

### Data source

This is a cross-sectional analysis using data from the CRONICAS Cohort Study [9] and the PREVENCION Study [10]. Cross-sectional data from the third follow-up round of the CRONICAS Cohort Study, conducted in year 2013-2014, was used to develop the risk score. Data from another population-based study, the PREVENCION Study (year 2004-2006), was used for external validation of the risk scores.

### Study population

The sample of the CRONICAS Cohort Study [9] was drawn from four settings in Peru: Lima (highly-urbanized city at sea level), Puno (including a rural and urban setting at 3825 m above the sea level) and Tumbes (semi-urban setting at sea level). This is a population-based study including subjects selected following a sex- and age-stratified (35-44, 45-54, 55-64, and ≥ 65 years) procedure. In each site 1000 subjects were enrolled. The sample included subjects whom were full-time residents in the area and capable of giving informed consent. Only one subject was recruited per household and the exclusion criteria included: being pregnant, having active pulmonary tuberculosis, and having any disability preventing them from undergoing anthropometric assessments. Further details about the sampling methods and procedures of the CRONICAS Cohort Study are available elsewhere [9].

For the development of the CKD score the initial sample included 2655 subjects, after excluding subjects with missing values in the prediction variables there were 2420 individuals. We further excluded subjects who reported having CKD (*N* = 14), because our risk score was for undiagnosed CKD; we also excluded subjects with missing values in key variables to calculate the eGFR (creatinine, age, sex and race), leaving a total of 2407 subjects. Lastly, we excluded subjects with BMI >40 kg/m^2^ or BMI <18.5 kg/m^2^, because extreme body mass can affect serum creatinine levels. Overall, 2368 subjects were included to develop the risk score (Additional file 1: Figure S1).

The PREVENCION Study [10] is a population-based study conducted in Arequipa, the second largest city in Peru at 2335 m above the sea level. The sample was selected following a probabilistic multistage sampling process, stratifying the sampling frame by socio-economic status and geographic location. Socio-economic status stratification was based on indicators of household sanitation and availability of urban services; regarding stratification by geographic location, the city was divided in areas of approximately 50 blocks and each of these further divided in 4-5 aggregates of approximately 150 households each. The PREVENCION researchers aimed to include ≥1600 subjects with at least 200 individuals in each age group: 20-34, 35-49, 50-64 and 65-80 years. Further details about the sampling methods and procedures of the PREVENCION Study have been published elsewhere [10].

In the validation process of the CKD scores there were initially 2106 individuals, we then excluded subjects who reported having the diagnosis of CKD (*n* = 3), and those with missing values in the prediction variables, resting 2024 individuals. In order to ensure comparability between the two studies, we excluded subjects aged <35 years in the PREVENCION Study, so there were 1611 subjects left. We further excluded subjects with missing values in key variables to calculate the eGFR and people with BMI < 18.5 kg/m^2^ or BMI > 40 kg/m^2^, leaving a total sample of 1459 subjects.

### Variables

For comparison purposes potential risk factors were defined similarly in both, the CRONICAS and PREVENCION studies. Table 1 provides detailed definitions used for various risk factors. Potential risk factors included clinical- and laboratory-based variables and all were assessed as potential risk factors in the development process [4]. Information was collected by trained fieldworkers through face-to-face interviews, and blood pressure and anthropometric indicators, i.e. weight and height, were also measured. Blood pressure measurements were conducted according to the recommendations of the 7th Joint National Committee on the diagnosis and management of High Blood Pressure in adults (JNC-7) [11]. Serum Creatinine was analysed from blood samples withdrawn from each participant. The CRONICAS and PREVENCION studies followed standardized procedures detailed elsewhere [9, 10].

**Table 1:** Variables definition in the CRONICAS Cohort Study and PREVENCION Study

Variable	
Age	< 50, 50-69, ≥ 70 years.
Sex	Men or women.
Self-history of cardiovascular disease	Either heart attack, stroke or heart failure: yes or no.
Smoking^a^	*Last 12 months, have you smoked?* Yes or no.
Hypertension	Defined as blood pressure ≥ 140/90 mmHg OR previous diagnosis of hypertension and currently under treatment [11].
Diabetes	Defined as fasting glucose ≥ 126 mg/dL OR diabetes diagnosed by a physician and currently under treatment.
Body Mass Index (BMI)	Categories: normal weight as [18.5-25[; overweight as [25-30[; and obesity as ≥ 30Kg/m^2^.
Central obesity	Waist circumference ≥ 90 cm if male or ≥80 cm if female.
Parental history of early heart attack	Parents with heart attack before age 60: yes or no.
Anemia	Haemoglobin < 13 g/dL if male and < 12 g/dL if female [24].
Total Cholesterol	Borderline high or high if total cholesterol ≥200 mg/dL versus desirable if total cholesterol <200 md/dL [25].
HDL Cholesterol	High if ≥ 50 mg/dL if female or ≥ 40 mg/dL if male versus low otherwise [25].
LDL Cholesterol	Above optimal or high if LDL cholesterol ≥ 100 mg/dL versus optimal otherwise [25].
Triglycerides	High > 150 mg/dL or low ≤ 150 mg/dL [25].

^a^Smoking was not defined in the exact same way, due to data availability: in PREVENCION it was defined as current smoker (yes or no)

The main outcome was CKD defined as an eGFR <60 mL/min/1.73m^2^ [12], using the MDRD (Modification of Diet in Renal Disease) formula, also known as CKD stage III [13]. For sensitivity analysis, the risk scores for prevalent undiagnosed CKD were also tested with the GFR estimated with the CKD-EPI (Chronic Kidney Disease Epidemiology Collaboration) formula specific for sex and race [14].

### Statistical analysis

#### Overall approach

Analyses were conducted with STATA 13.0 (StataCorp, College Station, TX, US). First, characteristics of the study population were summarized using means and standard deviations (SD) for numeric variables. Categorical variables were summarized using percentages and counts. We used the Chi-squared test to compare differences between groups. We developed two risk scores for prevalent undiagnosed CKD: complete and a laboratory-free risk score. The complete model included all associated risk factors (clinical- or laboratory-based variables), and a laboratory-free approach was pursued excluding information from blood test.

#### Development of the risk score

The development process was conducted with the CRONICAS dataset. Potential risk factors were included in bivariate models using logistic regressions, with the outcome being CKD; results were expressed as log of Odds Ratios (OR) and OR with 95% Confidence Intervals (95% CI). Risk factors with a *P*-value < 0.20 were included in the multivariable model. When all risk factors were included, we used a stepwise backward elimination technique until only significant risk factors, at a p-value < 0.05, remained in the model. To evaluate how accurate was the predicted prevalence of CKD relative to the observed prevalence, we used the Hosmer-Lemeshow test [15].

Each category of the risk factors included in the final model is given a value; these should be added up to calculate the risk. These values were obtained by rounding up the regression coefficients of the multivariable model. To determine the optimal cut-off point for the risk scores, we used the Youden’s index [16].

#### Validation process

To assess the properties of the risk scores –complete and laboratory-free– we calculated the area under the receiver operating characteristic (ROC) curve, sensitivity, specificity, positive/negative predictive values, percentage of correctly classified, and positive/negative likelihood ratios.

We internally validated the performance of our risk scores with bootstrap procedures. We used 1000 random samples with replacement to calculate the bootstrap ROC confidence interval choosing the bias-corrected option [17]. This procedure was conducted with CRONICAS dataset. On the other hand, the external validation, in terms of area under ROC, sensitivity, specificity, negative/positive predictive values as well as negative/positive likelihood ratios, was conducted with PREVENCION dataset.

Finally, we compared the performance of our risk scores –complete and laboratory-free– with those previously developed in other populations [4, 18–20]. These comparisons were made by contrasting the areas under the curves at the corresponding cut-off points. A recent systematic review [4] compiled several risk scores for CKD, of these, we chose those developed with cross-sectional studies and that assessed CKD in a similar fashion [18–20]. Although we were deficient of one or two predictors used in such risk scores, we strongly felt it was necessary to compare our risk scores to see if they were superior to existing ones.

#### Sensitivity analysis

The procedures to develop the risk scores were *re-conducted* including different parameters: i) the outcome, CKD, was estimated with the CKD-EPI equation; and ii) using the MDRD equation to estimate the eGFR, subjects with an eGFR < 15 (CKD Stage V) were excluded of the models. Because there are no current recommendations as to which equation use in Peru, we aimed to test our risk score using two different equations to verify if our risk scores could be used regardless of how eGFR was estimated. Then, we excluded subjects with CKD Stage V because these subjects could have other conditions than subjects with eGFR between 15 and 60, thus biasing our results; in addition, these patients would have a condition severe enough that a risk prediction tool would be unnecessary.

Other sensitivity analysis we conducted when developing the risk scores included adjusting the bivariate model by study site (as a proxy of altitude above the sea level). We aimed to see if the association was independent of geographic location, providing the study sites were at sea level and at high altitude.

#### Ethics

All participants in the CRONICAS Cohort Study gave informed consent and the study protocol was approved the Institutional Review Boards (IRB) at Universidad Peruana Cayetano Heredia (Lima, Peru) and Johns Hopkins University (Baltimore, USA) [9]. Participants in the PREVENCION Study gave informed consent and the study protocol was approved by the Santa Maria Catholic University Human Research Committee (Arequipa, Peru) [10].

## Results

### Characteristics of the participants

Participants in both studies were 57.7 years old in average, and over 50% were females. There were differences in mean BMI and lipid profiles: BMI was higher in CRONICAS while total cholesterol, HDL-cholesterol and triglycerides were higher in PREVENCION. Further details about each study population are depicted in Table 2.

**Table 2:** Sociodemographic and clinical characteristics of the participants

	CRONICAS	PREVENCION	*P*-value
	(*n* = 2368)	(*n* = 1459)	
Sex			
Women (%)	50.6	52.3	0.31
Age^a^			
Mean (SD)	57.7 (12.4)	57.1 (12.6)	0.20
Smoke			
Yes (%)	12.1	16.2	<0.01
Diabetes			
Yes (%)	8.4	6.7	0.06
Fasting Glucose			
Mean (SD)	104.1 (42.3)	84.8 (27.8)	<0.01
Hypertension^a^			
Yes (%)	24.0	22.8	0.39
Systolic Blood Pressure			
Mean (SD)	118.6 (19.7)	123.3 (20.2)	<0.01
Diastolic Blood Pressure			
Mean (SD)	72.6 (10.8)	79.2 (9.1)	<0.01
Personal History of CVD			
Yes (%)	0.9	2.6	<0.01
Anemia^a^			
Yes (%)	8.1	2.7	<0.01
Haemoglobin			
Mean (SD)	14.5 (2.1)	15.1 (1.7)	<0.01
BMI			
Mean (SD)	27.9 (4.1)	27.2 (4.0)	<0.01
Waist Circumference			
Mean (SD)	93.0 (10.1)	92.8 (10.9)	0.51
Total Cholesterol			
Mean (SD)	197.8 (40.5)	206.7 (39.2)	<0.01
HDL-Cholesterol			
Mean (SD)	45.7 (12.7)	47.9 (10.1)	<0.01
LDL-Cholesterol			
Mean (SD)	120.7 (34.4)	122.6 (31.5)	0.09
Triglycerides			
Mean (SD)	158.6 (92.9)	184.0 (91.3)	<0.01

All these risk factors were assessed in the bivariate model

^a^Indicates risk factors included in the final adjusted model

### Prevalence of undiagnosed CKD

In CRONICAS, according to the MDRD equation, mean eGFR was 96.3 (SD: 21.6) and there was a CKD Stage III or greater prevalence of 3.4% (95% CI: 2.7%-4.2%). In PREVENCION, mean eGFR was 89.6 (SD: 20.1) and there was a CKD Stage III or greater prevalence of 5.4% (95% CI: 4.3%-6.6%). Mean eGFR (*P* < 0.001) and the CKD prevalence was different between studies (*P* = 0.003).

### CRONICAS-CKD risk score: Development

Table 3 shows risk factors associated with undiagnosed CKD, both in the univariable and multivariable models. Risk factors strongly associated with prevalent undiagnosed CKD in the multivariable model were age, hypertension and anemia. Age showed the strongest OR: older age was associated with higher odds of having impaired eGFR. Likewise, having hypertension and anemia was associated with almost 5-fold and 4-fold higher odds of having CKD, respectively.

**Table 3:** Associated factors with undiagnosed CKD: Beta Coefficients and Odds Ratios using CRONICAS database (*N* = 2368)

	Univariable Model		Multivariable Model		Risk Score (points)
	Coefficient (SE)	OR (95%CI, *P*-value)	Coefficient (SE)	OR (95%CI, *P*-value)	
Sex					
Women	1	1			
Men	0.18 (0.23)	1.20 (0.76-1.87, 0.45)			
Age					
< 50	1	1	1	1	0
50-69	1.82 (0.61)	6.17 (1.87-20.32, < 0.01)	1.54 (0.61)	4.66 (1.40-15.53, 0.01)	1
≥ 70	3.48 (0.60)	32.30 (10.00-104.40, < 0.01)	2.59 (0.61)	13.38 (4.01-44.62, < 0.01)	2
Personal History of any CVD					
No	1	1			
Yes	1.59 (0.63)	4.91 (1.42-17.09, 0.01)			
Personal History of Infarction					
No	1	1			
Yes	−0.72 (1.04)	0.49 (0.06-3.75, 0.49)			
Personal History of Stroke					
No	1	1			
Yes	−3.38 (1.01)	0.03 (0.01-0.25, < 0.01)			
Personal History of Heart Failure					
No	1	1			
Yes	−2.67 (1.23)	0.07 (0.01-0.77, 0.03)			
Smoking					
No	1	1			
Yes	−0.74 (0.47)	0.48 (0.19-1.19, 0.11)			
Hypertension					
No	1	1	1	1	0
Yes	2.15 (0.25)	8.60 (5.25-14.09, < 0.01)	1.54 (0.27)	4.65 (2.76-7.83, < 0.01)	1
Diabetes					
No	1	1			
Yes	0.80 (0.29)	2.22 (1.26-3.90, < 0.01)			
BMI					
Normal	1	1			
Overweight	−0.22 (0.27)	0.80 (0.47-1.37, 0.42)			
Obesity	−0.20 (0.30)	0.82 (0.45-1.48, 0.50)			
Central Obesity					
No	1	1			
Yes	0.29 (0.30)	1.34 (0.75-2.40, 0.33)			
Parents w/Infraction <60y					
No	1	1			
Yes	−0.74 (0.72)	0.48 (0.12-1.96. 0.30)			
Anemia					
No	1	1	1	1	0
Yes	1.82 (0.25)	6.16 (3.76-10.09, < 0.01)	1.27 (0.28)	3.56 (2.07-6. 21, < 0.01)	1
Total Cholesterol					
Desirable	1				
Borderline High/High	0.31 (0.23)	1.36 (0.87-2.13, 0.18)			
HDL-Cholesterol					
Low	1	1			
High	−0.08 (0.23)	0.92 (0.59-1.44, 0.71)			
LDL-Cholesterol					
Optimal	1	1			
Above Optimal/High	−0.16 (0.25)	0.85 (0.53-1.38, 0.52)			
Triglycerides					
≤ 150	1	1			
> 150	0.28 (0.23)	1.32 (0.85-2.07, 0.22)			

The multivariable model was created following a backward elimination approach: all variables with *p* < 0.20 in the univariable model were fitted and one by one removed starting with the least significant until the final model only included significant variables (*p* < 0.05). Although personal history of stroke or personal history of heart failure could have been included in the multivariable model, these were not included because their estimates were very small and personal history of any CVD, which included the aforementioned conditions, had a higher and significant estimate. For the final multivariable model: Hosmer-Lemeshow X^2^ test: 4.13 with a p-value of 0.53. Area under de ROC curve of the final model was 0.8445. Each category of the risk factors included in the final model is given the corresponding point (see last column), and add up. For both, the complete and laboratory-free risk scores, the optimal cut-off point was 2 points: if a subject reaches 2+ points he/she would have greater odds of having CKD at that time; for example a 75-year old men with hypertension but without anemia would score 3 (2 points of age and 1 for hypertension)

**Fig. 1:**
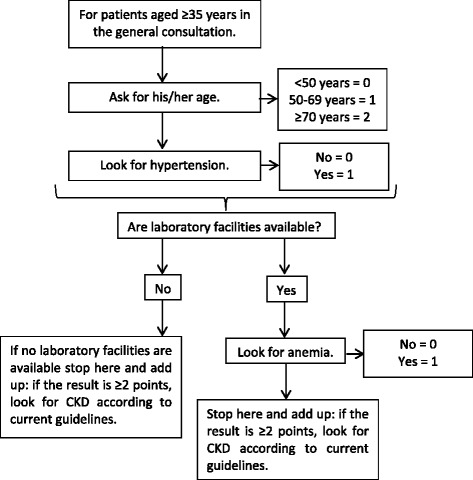
Algorithm to use the CRONICAS-CKD risk score in the general population

Regarding the scoring system, for age there were three categories: <50 years (0 points), 50-69 (1 point) and ≥ 70 years (2 points); with regards to hypertension, there were two categories: no (0 points) and yes (1 point); and anemia also had two categories: no (0 points) and yes (1 point). Therefore, the complete risk score could add up to 4 points, whereas the laboratory-free (without anemia) risk score could add up to 3 points. For both, the complete and laboratory-free risk scores, the optimal cut-off point was 2 points (Fig. 1).

**Fig. 2:**
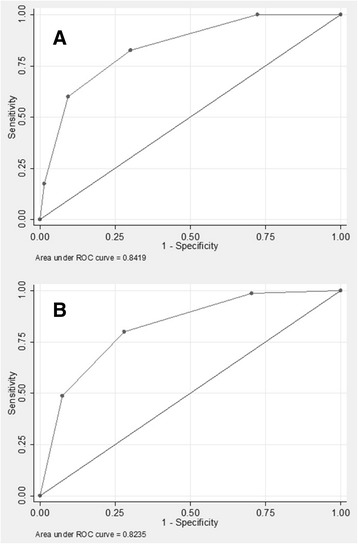
Receive operating characteristic (ROC) curve of the CRONICAS-CKD risk score for undiagnosed CKD using CRONICAS database. (**a**) complete risk score, the 95% CI of the area under the ROC curve is 79.6%-88.0%; (**b**) laboratory-free risk score, the 95% CI of the area under the ROC curve is 77.7%-86.8%. The areas between these curves were different (*P* = 0.028)

Table 4 depicts the performance of the complete and laboratory-free risk scores; in addition, Fig. 2 shows the area under the ROC curve for the complete and laboratory-free risk score: 0.842 and 0.824 (*P* = 0.03), respectively. At a cut-off point of 2, the areas under the curve were not different between the complete and laboratory-free risk scores: 76.2% versus 76.0% (*P* = 0.78).

**Table 4:** Performance of the CRONICAS-CKD risk score for undiagnosed CKD using CRONICAS dataset

Total score	Youden Index	Sensitivity	Specificity	PPV	NPV	Correctly classified	LR+	LR-
	Complete Risk Score							
≥ 0	0%	100.0%	0.0%	3.4%	–	3.4%	1.00	–
≥ 1	27.7%	100.0%	27. 7%	4.6%	100.0%	30.1%	1.4	0.00
*≥ 2*	*52.5%*	*82.5%*	*70.0%*	*8.8%*	*99.1%*	*70.4%*	*2.8*	*0.3*
≥ 3	50.7%	60.0%	90.7%	18.3%	98.5%	89.6%	6.4	0.4
≥ 4	16.1%	17.5%	98.6%	29.8%	97.2%	95.8%	12.1	0.8
	laboratory-free Risk Score							
≥ 0	0%	100.0%	00.0%	3.4%	–	3.4%	1.0	–
≥ 1	28.3%	98.8%	29.6%	4.7%	99.9%	31.9%	1.4	0.0
*≥ 2*	*52.0%*	*80.0%*	*72.0%*	*9.1%*	*99.0%*	*72.3%*	*2.9*	*0.3*
≥ 3	41.3%	48.8%	92.6%	18.7%	98.1%	91.1%	6.6	0.6

*PPV* Positive predictive value, *NPV* Negative predictive value, *LR+* Positive likelihood ratio, *LR-* Negative likelihood ratio

PPV and NPV were calculated with a CKD prevalence of 3.38%. At a cut-off point of 2, the areas under the ROC were similar between the complete and laboratory-free risk scores (76.24% versus 75.99%, *P* = 0.784). The italicized figures highlight the characteristics at the proposed cut-off point

### CRONICAS-CKD risk score: External validation

Using PREVENCION dataset, the complete risk score with an optimal cut-off point of 2 showed a sensitivity of 70.5%, a specificity of 69.1%, a positive predictive value of 11.4%, a negative predictive value of 97.6%, a positive likelihood ratio of 2.3 and a negative likelihood ratio of 0.4; this yielded a ROC area of 70.0%. The laboratory-free risk score, at a cut-off point of 2, showed a sensitivity of 70.5%, a specificity of 69.7%, a positive predictive value of 11.6%, a negative predictive value of 97.7%, a positive likelihood of 2.3 and a negative likelihood of 0.4; this yielded a ROC are of 70%. Of note is the high negative predictive value of the complete and laboratory-free risk scores.

### Comparison with other risk scores

At the corresponding threshold points, when ours risk scores were compared with others, these were very similar in terms of c-statistics. In addition, the ROC area was not different between our risk scores and the others, except when comparing our complete risk score with that one from Thailand: 76.2% (ours) versus 72.2% (*P* = 0.03). Further details about these comparisons are presented in Additional file 2: Table S1.

### Sensitivity analysis

Additional file 3: Table S2 shows the univariable and multivariable models when the outcome was defined using the CKD-EPI equation. If a risk score for undiagnosed CKD were to be constructed with eGFR defined with the CKD-EPI equation, such risk score should also include personal history of any cardiovascular disease in addition to age, hypertension and anemia.

Additional file 4: Table S3 shows the univariable and multivariable models when subjects with eGFR (according to the MDRD equation) less than 15 were excluded. If the risk score were to be developed for subjects with eGFR >15 only, it should also include triglycerides besides age, hypertension and anemia.

When the bivariate associations were further adjusted by study site (as a proxy for altitude above the sea level), the results did not change, signalling that the associations seemed to be independent of geographic location.

## Discussion

### Main results

In Peru, an algorithm to identify subjects aged 35+ years in the general population with prevalent undiagnosed CKD, defined as an eGFR <60 ml/min/1.73m^2^ according to the MDRD equation, should include age of the patient, as well as hypertension and anemia status. Furthermore, if no laboratory facilities are available, just including age and hypertension status is a sensitive and specific approach too. The risk score herein presented should be used with the GFR estimated using the MDRD equation and including subjects with eGFR in any range. The risk scores are relevant in the primary care level or in rural settings where subjects with a negative result in the risk score may not need further testing; nonetheless, a positive result would require further exploration. However, because these findings depend on the condition’s prevalence, they should be interpreted with caution in each setting. Since previous risk scores have not been developed exclusively with populations from Latin America or the Andes region, we offer a practical tool to discard prevalent undiagnosed CKD in these populations.

### Interpretation of results

The positive and negative predictive values depend on the prevalence of the condition; the low CKD prevalence in the study population could explain the low positive predictive value. Furthermore, we reported high negative predictive values, meaning that, of all tested subjects who had a negative result, over 99% do not have CKD. This is a good feature because subsequent tests could be expensive, and our risk scores show that subjects with negative results are likely not to have CKD, and thus may not need to undergo further testing. However, negative and positive predictive values depend on the prevalence of the condition. Thus, interpretation of these figures should be made with caution and according to local epidemiology.

Moreover, since the area under the ROC curves were not different between the complete and laboratory-free scores at a threshold of 2 points, this suggests that the risk scores could be interchangeably, prioritizing the laboratory-free risk score because it is a much easier approach. Overall, given the negative predictive value is higher than the positive predictive value, we suggest using the risk scores as a screening tool discard CKD; however, a positive result would need further confirmation.

When comparing ours risk score with others similarly developed, the results showed there were not strong differences. This suggests ours risk scores perform as well as those previously developed [18–20]. Nonetheless, ours have fewer variables, particularly the laboratory-free risk score. This accounts for its simplicity and easy use, which are valuable features in resource-limited settings where clinically-assessed variables may be the only available tools.

### Comparison of results

The fact that our risk scores perform similarly to those previously developed (Additional file 2: Table S1) could be because the risk scores included a similar base: age, hypertension and anemia status. This highlights the relevance of these variables to identify CKD. However, the higher or lower ROC area could be because other risk scores included more predictors such as proteinuria, self-history of kidney stones, and peripheral vascular diseases, among others; some of these variables are laboratory-based, restraining their availability in resource-limited or rural areas. Although we did not assess these parameters, our risk scores still presented a moderate performance.

### Limitations and opportunities of using previous CKD risk scores

Although some of the previous risk scores claimed to have been developed in multi-ethnic populations [4], none was developed mainly including subjects from Latin America or the Andes region. Even though subjects with Hispanic or Latino background in the USA have lower rates of eGFR < 60 relative to Mexican Americans, whites and black individuals, when stratified by sex, males have similar rates across these ethnicities and when stratified by age, Hispanic/Latinos aged 45-54 years have higher rates [21]. Thus probably, Hispanic and Latinos need to be screened differently than native Americans. Because international and internal migration keeps growing, it becomes relevant to have specific tools to accurately identify subjects at increased CKD risk. These specific tools could be used at medical appointments based on the patient’s ethnic background; for example, patients from the Latin America or Andes region could be assessed with our risk scores, probably providing more accurate results than if they had been assessed with other scores. Notwithstanding, this warrants further verification.

### Relevance and implications

The risk scores herein presented, included age, that could be assessed by anyone with a questionnaire; blood pressure, that could be assessed by any one with little training and minimal supervision; and anemia status, for which blood samples can be taken by anyone with minimal training and there are also point-of-care options. Thus, our risk scores could be self-administered or applied by community-health workers. Community-health workers seem to be a cost-effective approach in LMICs, although this approach has been mostly assessed in the field of communicable diseases, maternal and new born health [22]. Our results, along with others in the literature, could be used to assess how community-health workers identify and adequately refer subjects with undiagnosed CKD. According to our risk score, 50+ year old subjects with hypertension should be looked up for CKD; therefore, health personnel or community-health workers could identify and refer these subjects.

The Peruvian Society of Nephrology suggests that at the primary care level a general physician should identify subjects with risk factors for CKD. In addition, they suggest to take a complete urine test looking for proteinuria and also to assess micro-albuminuria [23]. Unfortunately, these tests may not be available everywhere, or may not be affordable. Furthermore, and although urine dipsticks could also be an option, securing their provision across the country would face major setbacks too, limiting their application. Thus, our risk score could provide an additional filter to classify subjects who should undergo further examinations. Nevertheless, our risk score should be further studied before strong use recommendations are made.

### Strengths and limitations

We used two different datasets for internal and external validation. Furthermore, the data we used included subjects of four different Peruvian cities, encompassing rural and urban settings, as well as different geographic profiles. Although the data was not nationally representative, the broad range of participants could make our results informative and applicable to other Peruvian settings, and to other settings with similar characteristics in the Latin American or Andes region.

Nevertheless, limitations must be highlighted. First, we did not include other potential risk factors assessed by previous risk scores, including albumin excretion or proteinuria. Second, our CKD definition was only based on eGFR, although other parameters could have been included (e.g., albuminuria or structural abnormalities in the kidney) [5]. This limitation is shared with other risk scores [4]. Although a previous study in Peru reported that proteinuria was the most common criterion for CKD [3], its assessment is not available everywhere, thus not fulfilling our main goal: to develop an easy-to-apply risk score for prevalent undiagnosed CKD, which could be used in any medical facility regardless of the availability of laboratory facilities. Furthermore, because albuminuria is an important predictor of decline in eGFR, subjects at high risk in our scores could undergo such test, and not before, saving resources. Third, including elderly people (e.g. subjects aged ≥70 years) could have seen as a limitation, because many would have low eGFR and multi-morbidity accounting for the low specificity of the risk scores. Fourth, we could not define the etiology of anemia. This could account for the low specificity of the complete risk score, because a 50-year-old person with anemia due to blood loss would be classified as at higher risk of CKD. Finally, we did not have access to the participants’ medical records, which could have been useful to identify more CKD patients who, due to any reasons, may have been unaware of their condition when they were invited to participate in the studies. However, because health care is mostly curative-oriented, we believe a patient who underwent creatinine evaluation (or any other test to assess kidney function) without being informed of his/her condition, would be extremely rare. This scenario also assumes that laboratory facilities are widely available, which is not true; on this fact relies the importance of our risk scores, because it would allow identifying high-risk subjects who should be then referred to a laboratory facility.

## Conclusions

Including hypertension and age in a risk score is a useful first approach to screen for prevalent undiagnosed CKD; adding anemia status to these variables did not improve much the performance of the risk score meaning that it is not compulsory to have laboratory facilities to apply the risk score. In LMIC, where laboratory facilities are still scarce, pragmatic approaches, as the ones herein described, could be a useful screening tool to identify cases of CKD and thus prevent its great health burden in terms of morbidity and mortality.

## Additional files

Additional file 1: Figure S1. Flow-chart of participants included from the CRONICAS Cohort Study in the development of the risk score. (DOCX 18 kb)

Additional file 2: Table S1. Comparison with other risk scores. (DOCX 14 kb)

Additional file 3: Table S2. Regression models with undiagnosed CKD defined using the CKD-EPI equation in CRONICAS dataset (sensitivity analysis, *N* = 2368). (DOCX 17 kb)

Additional file 4: Table S3. Regression models with undiagnosed CKD having excluded subjects with CKD (as per MDRD equations) < 15 using the CRONICAS database (sensitivity analysis, *N* = 2364). (DOCX 17 kb)
