# Supplementary material for: Risk score for first-screening of prevalent undiagnosed chronic kidney disease in Peru: the CRONICAS-CKD risk score
Source: BMC Nephrol. 2017 Nov 29;18:343. doi: 10.1186/s12882-017-0758-4 (PMC5707893; doi:10.1186/s12882-017-0758-4)
Supplement: Supplementary file 1 — Flow-chart of participants included from the CRONICAS Cohort Study in the development of the risk score. (DOCX 18 kb) [file 12882_2017_758_MOESM1_ESM.docx]

## Supplementary Figure 1: Flow-chart of participants included from the CRONICAS Cohort Study in the development of the risk score

CRONICAS Cohort Study 3rd Follow-Up Round

N=2,726

Matched subjects after merging variables from previous follow-up rounds

N=2,655

After excluding subjects with missing values in assessed potential risk factors

N=2,420

After excluding subjects who reported having had the diagnosis of CKD at the 1^st^ or 2^nd^ follow-up round

N=2,407

After excluding subjects with BMI >40 or BMI <18.5

N=2,368
