# Supplementary material for: Risk score for first-screening of prevalent undiagnosed chronic kidney disease in Peru: the CRONICAS-CKD risk score
Source: BMC Nephrol. 2017 Nov 29;18:343. doi: 10.1186/s12882-017-0758-4 (PMC5707893; doi:10.1186/s12882-017-0758-4)
Supplement: Supplementary file 2 — Comparison with other risk scores. (DOCX 14 kb) [file 12882_2017_758_MOESM2_ESM.docx]

## Table S1: Comparison with other risk scores.

| **SCORED Risk Score**  Bang H, Vupputuri S, Shoham DA, et al. SCreening for Occult REnal Disease (SCORED): a simple prediction model for chronic kidney disease. *Archives of internal medicine* 2007; 167(4): 374-81. | At its respective cut-off points, the SCORED risk score had a ROC area of 74.0%, a sensitivity of 86.3%, a specificity of 62.6%, a positive predictive value of 7.5%, a negative predictive value of 99.2%, and a positive and negative likelihood ratio of 2.3 and 0.2, respectively. Compared to our risk scores, at their corresponding cut-off points, the areas under the ROC were not different: 76.2% (ours) versus 74.4% (p=0.287). Likewise when compared to our laboratory-free risk score: 76.0% (ours) versus 74.4% (p=0.352). |
| --- | --- |
| **Thailand Risk Score**  Thakkinstian A, Ingsathit A, Chaiprasert A, et al. A simplified clinical prediction score of chronic kidney disease: a cross-sectional-survey study. *BMC nephrology* 2011; 12: 45. | The Thailand risk score did not provide a single unique cut-off point, yet they set a range of scores based on their values of positive likelihood ratio. However, based on their results, the highest Youden’s index would be at 6-8 points. Using this threshold (6+ points), the ROC area was 72%, the sensitivity of 88.8%, the specificity of 55.6%, the positive predictive value of 6.5%, the negative predictive value of 99.3%, the positive likelihood ratio of 2.0, and the negative likelihood of 0.2. Compared to our complete risk score, the area under the ROC was different: 76.2% (ours) versus 72.2% (p=0.034); whereas when compared with the laboratory-free risk score, there were not differences: 76.0% (ours) versus 72.2 (p=0.067). |
| **Korean Risk Score**  Kwon KS, Bang H, Bomback AS, et al. A simple prediction score for kidney disease in the Korean population. *Nephrology (Carlton, Vic)* 2012; 17(3): 278-84. | The Korean risk score, at a threshold of 4 points, revealed a ROC area of 74%, a sensitivity of 86.3%, a specificity of 62.6%, a positive predictive value of 7.5%, a negative predictive value of 99.2%, and a positive and negative likelihood ratio of 2.3 and 0.2, respectively. Compared to our risk scores at a cut-off point of 2, there were not differences: 76.2% (ours) versus 74.4% (p=0.287) relative to ours complete risk score, and 76.0% (ours) versus 74.4% (p=0.352) in comparison to our laboratory-free risk score. |
