# Supplementary material for: Risk score for first-screening of prevalent undiagnosed chronic kidney disease in Peru: the CRONICAS-CKD risk score
Source: BMC Nephrol. 2017 Nov 29;18:343. doi: 10.1186/s12882-017-0758-4 (PMC5707893; doi:10.1186/s12882-017-0758-4)
Supplement: Supplementary file 4 — Regression models with undiagnosed CKD having excluded subjects with CKD (as per MDRD equations) < 15 using the CRONICAS database (sensitivity analysis, N = 2364). (DOCX 17 kb) [file 12882_2017_758_MOESM4_ESM.docx]

## Table S3: Regression models with undiagnosed CKD having excluded subjects with CKD (as per MDRD equations) <15 using the CRONICAS database (sensitivity analysis, N=2,364).

|  | Univariable Model | | Multivariable Model | |
| --- | --- | --- | --- | --- |
|  | Coefficient (SE) | OR (95%CI, p-value) | Coefficient (SE) | OR (95%CI, p-value) |
| Sex |  |  |  |  |
| Women | 1 | 1 |  |  |
| Men | 0.29 (0.24) | 1.34 (0.85-2.13, 0.21) |  |  |
| Age |  |  |  |  |
| <50 | 1 | 1 | 1 | 1 |
| 50-69 | 2.23 (0.73) | 9.25 (2.20-38.88, <0.01) | 1.93 (0.74) | 6.86 (1.62-29.09, 0.01) |
| ≥70 | 3.82 (0.73) | 32.73 (10.96-188.28, <0.01) | 3.06 (0.74) | 21.29 (5.00-90.77, <0.01) |
| Personal History of any CVD |  |  |  |  |
| No | 1 | 1 |  |  |
| Yes | 1.23 (0.76) | 3.41 (0.78-14.96, 0.10) |  |  |
| Personal History of Infraction |  |  |  |  |
| No | 1 | 1 |  |  |
| Yes | -0.77 (1.04) | 0.46 (0.06-3.56, 0.46) |  |  |
| Personal History of Stroke |  |  |  |  |
| No | 1 | 1 |  |  |
| Yes | -2.72 (1.23) | 0.07 (0.01-0.73, 0.03) |  |  |
| Personal History of Heart Failure |  |  |  |  |
| No | 1 | 1 |  |  |
| Yes | -2.72 (1.23) | 0.07 (0.01-0.73, 0.03) |  |  |
| Smoking |  |  |  |  |
| No | 1 | 1 |  |  |
| Yes | -0.69 (0.47) | 0.50 (0.20-1.26, 0.14) |  |  |
| Hypertension |  |  |  |  |
| No | 1 | 1 | 1 | 1 |
| Yes | 2.14 (0.26) | 8.51 (5.14-14.11, <0.01) | 1.50 (0.27) | 4.49 (2.63-7.643, <0.01) |
| Diabetes |  |  |  |  |
| No | 1 | 1 |  |  |
| Yes | 0.69 (0.30) | 2.00 (1.10-3.63, 0.02) |  |  |
| BMI |  |  |  |  |
| Normal | 1 | 1 |  |  |
| Overweight | -0.13 (0.28) | 0.88 (0.51-1.52, 0.64) |  |  |
| Obesity | -0.21 (0.32) | 0.81 (0.44-1.51, 0.51) |  |  |
| Central Obesity |  |  |  |  |
| No | 1 | 1 |  |  |
| Yes | 0.32 (0.31) | 1.38 (0.75-2.52 0.30) |  |  |
| Parents w/Infraction <60y |  |  |  |  |
| No | 1 | 1 |  |  |
| Yes | -0.69 (0.72) | 0.50 (0.12-2.07, 0.34) |  |  |
| Anemia |  |  |  |  |
| No | 1 | 1 | 1 | 1 |
| Yes | 1.65 (0.27) | 5.21 (3.10-8.76, <0.01) | 1.18 (0.30) | 3.26 (1.83-5.82, <0.01) |
| Total Cholesterol |  |  |  |  |
| Desirable | 1 |  |  |  |
| Borderline High/High | 0.31 (0.23) | 1.37 (0.87-2.16, 0.18) |  |  |
| HDL-Cholesterol |  |  |  |  |
| Low | 1 | 1 |  |  |
| High | -0.19 (0.23) | 0.83 (0.52-1.31, 0.42) |  |  |
| LDL-Cholesterol |  |  |  |  |
| Optimal | 1 | 1 |  |  |
| Above Optimal/High | -0.11 (0.25) | 0.89 (0.54-1.47, 0.66) |  |  |
| Triglycerides |  |  |  |  |
| ≤150 | 1 | 1 | 1 | 1 |
| >150 | 0.33 (0.23) | 1.39 (0.88-2.19, 0.16) | 0.62 (0.25) | 1.87 (1.13-3.07, 0.01) |

The multivariable model was created following a backward elimination approach: all variables with p<0.20 in the univariable model were fitted and one by one removed starting with the least significant until the final model only included significant variables (p<0.05). Although personal history of stroke or personal history of heart failure could have been included in the multivariable model, these were not included because their estimates was very small and personal history of any CVD, which included the aforementioned conditions, had a higher and significant estimate. For the final mode: Hosmer-Lemeshow X^2^ test: 3.82 with a p-value of 0.70. Area under ROC curve of the final model was 0.8595.
